# Reduced Bacterial Counts from a Sewage Treatment Plant but Increased Counts and Antibiotic Resistance in the Recipient Stream in Accra, Ghana—A Cross-Sectional Study

**DOI:** 10.3390/tropicalmed6020079

**Published:** 2021-05-14

**Authors:** Lady A. B. Adomako, Dzidzo Yirenya-Tawiah, Daniel Nukpezah, Arpine Abrahamya, Appiah-Korang Labi, Ruzanna Grigoryan, Hawa Ahmed, Josiah Owusu-Danquah, Ted Yemoh Annang, Regina A. Banu, Mike Y. Osei-Atweneboana, Collins Timire, Hanock Tweya, Stephen E. D. Ackon, Emmanuel Nartey, Rony Zachariah

**Affiliations:** 1 CSIR-Water Research Institute, 2nd CSIR Close, Achimota, Accra P.O. Box AH 38, Ghana; Hawaahmed360@yahoo.com (H.A.); reginamabanu@gmail.com (R.A.B.); oseiatweneboana@yahoo.co.uk (M.Y.O.-A.); 2 Institute for Environmental and Sanitation Studies, University of Ghana, Accra P.O. Box LG 209, Ghana; dzidzoy@staff.ug.edu.gh (D.Y.-T.); dnukpezah@staff.ug.edu.gh (D.N.); niiyemoh@staff.ug.edu.gh (T.Y.A.); 3 TB Research and Prevention Center, Yerevan 0014, Armenia; arpine_abrahamyan@yahoo.com (A.A.); ruzanna.grigory@gmail.com (R.G.); 4 WHO Country Office, Accra P.O. Box LG 209, Ghana; labia@who.int; 5 Civil and Environmental Engineering Department, Cleveland State University, Cleveland Ohio, 2121 Euclid Ave. FH 121, Cleveland, OH 4411, USA; j.owusudanquah@csuohio.edu; 6 International Union Against Tuberculosis and Lung Disease, 68 Boulevard Saint-Michel, 75006 Paris, France; collins.timire@theunion.org (C.T.); hannock.tweya@gmail.com (H.T.); 7 Accra Sewerage Improvement Project/Accra Metro Sewerage Unit, Ministries, Accra P.O. Box MB 201, Ghana; ekowackon@yahoo.com (S.E.D.A.); kentkaya@gmail.com (E.N.); 8 UNICEF, UNDP, World Bank, WHO, Special Programme for Research and Training in Tropical Diseases, 20 Avenue Appia, 1211 Geneva, Switzerland; zachariahr@who.int

**Keywords:** SORT IT, One Health, operational research, sustainable development goals, antimicrobial resistance, antibiotic residues, wastewater treatment

## Abstract

Wastewater treatment plants receive sewage containing high concentrations of bacteria and antibiotics. We assessed bacterial counts and their antibiotic resistance patterns in water from (a) influents and effluents of the Legon sewage treatment plant (STP) in Accra, Ghana and (b) upstream, outfall, and downstream in the recipient Onyasia stream. We conducted a cross-sectional study of quality-controlled water testing (January–June 2018). In STP effluents, mean bacterial counts (colony-forming units/100 mL) had reduced *E. coli* (99.9% reduction; 102,266,667 to 710), *A. hydrophila* (98.8%; 376,333 to 9603), and *P. aeruginosa* (99.5%; 5,666,667 to 1550). Antibiotic resistance was significantly reduced for tetracycline, ciprofloxacin, cefuroxime, and ceftazidime and increased for gentamicin, amoxicillin/clavulanate, and imipenem. The highest levels were for amoxicillin/clavulanate (50–97%) and aztreonam (33%). Bacterial counts increased by 98.8% downstream compared to the sewage outfall and were predominated by *E. coli*, implying intense fecal contamination from other sources. There was a progressive increase in antibiotic resistance from upstream, to outfall, to downstream. The highest resistance was for amoxicillin/clavulanate (80–83%), cefuroxime (47–73%), aztreonam (53%), and ciprofloxacin (40%)**.** The STP is efficient in reducing bacterial counts and thus reducing environmental contamination. The recipient stream is contaminated with antibiotic-resistant bacteria listed as critically important for human use, which needs addressing.

## 1. Introduction

The increased production and use of antibiotics have resulted in “antibiotic residues” entering the environment through human, animal, and agricultural wastes [1]. Such residues promote the development of antibiotic resistance in common bacteria present in the environment such as *Escherichia coli* (*E. coli*), *Aeromonas hydrophila* (*A. hydrophila*), and *Pseudomonas aeruginosa* (*P. aeruginosa*) [1,2,3]. Then, this resistance may be transferred to other bacteria [4,5]. Human and animal waste deposited into the environment may also contain antibiotic-resistant bacteria. Such bacteria can move from one human to another or one animal to another via direct contact with water contaminated with resistant bacteria, or through the consumption of contaminated food, so-called “farm-to-fork” transmission in humans. 

Wastewater treatment plants receive sewage containing high concentrations of bacteria and antibiotics, particularly if they receive human and hospital effluents. Such treatment plants serve as excellent hotspots for continued bacterial replication and the transfer of antibiotic resistance between bacteria. The capacity of municipal sewage treatment plants to remove bacteria and antibiotic residues is variable depending on the methods used. This may result in high bacterial loads (counts) of resistant bacteria being discharged into water streams even after treatment [6,7]. 

The World Health Organization’s (WHO) global action plan to tackle antimicrobial resistance (AMR) emphasizes the “One Health” approach. This approach includes humans, animals, the environment, the food chain, and the interconnections between them as one entity [8]. In Ghana, most AMR surveillance activities have focused mainly on human and animal health and less on the environment [9], with evidence of increasing levels of AMR in both sectors [10,11].

The Legon sewage treatment plant in Accra, Ghana was constructed in 2012 as part of the Accra Sewerage Improvement Project funded by the African Development Bank. This plant operates on the concept of treatment of waste stabilization ponds and treats wastewater from various academic institutions and importantly from the Achimota Hospital. 

**Figure 1:**
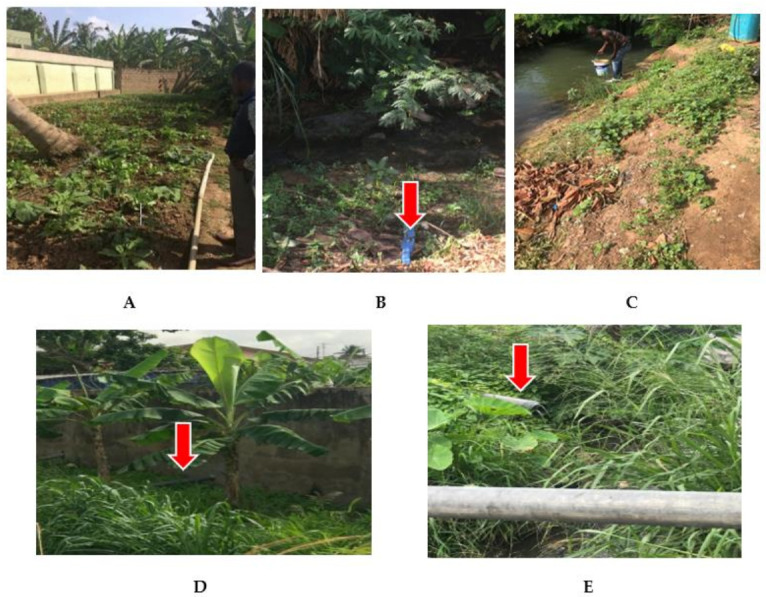
Onyasia stream showing a lettuce farm along the banks of the Onyasia stream (**A**) water being abstracted for irrigation (**B**,**C**), and domestic waste discharge into the stream (**D**,**E**).

Effluents from this plant are discharged directly into the Onyasia Stream [12]. The stream runs through a suburb of Accra and is used to irrigate farms, including lettuce and cabbage gardens (Figure 1) [13]. The water is also used for other purposes such as washing cars. Thus, resistant bacteria, if present in the stream, would “connect” with humans and animals resulting in the possible spread of any resistant pathogens [14,15]. 

Multidrug-resistant *E. coli* and *P. aeruginosa* are common bacteria, which can cause deadly human infections; thus, they are considered priority pathogens by the WHO for global surveillance of AMR. They are also less fastidious to grow in the laboratory and can serve as proxy organisms for measuring antibiotic resistance circulating in the environment [16]. *A. hydrophila* is an aquatic bacterium that causes disease in fish and amphibians such as frogs and is often abundant in wastewater. *A. hydrophila* may cause gastroenteritis and cholera-like illness [17]. 

A PubMed search revealed no study from West Africa assessing the role of treatment plants in reducing the loads of these three bacteria and their antibiotic resistance profiles. Comparing bacterial counts and antibiotic resistance profiles in influent and effluent wastewater from the Legon treatment plant will provide an understanding of the efficiency of the sewage treatment process. Furthermore, the level of antibiotic resistance in sewage effluents can serve as a “proxy” for levels of antibacterial resistance circulating in the environment. We hypothesize that sewage outfall will be diluted in the Onyasia stream, and hence, there will be a reduction in bacterial counts further downstream. 

Our specific objectives were to assess the bacterial counts of *E. coli*, *P. aeruginosa*, and *A. hydrophila* and their antibiotic resistance profiles in influent and effluent wastewater from the Legon sewage treatment plant and at upstream, sewage outfall, and downstream points in the Onyasia stream. 

## 2. Materials and Methods

### 2.1. Study Design

This is a cross-sectional study using secondary laboratory data on water samples.

### 2.2. Study Setting

Ghana lies in West Africa, and the capital is Accra. The country has a population of about 30 million, of which about 2.3 million live in Accra [18]. Currently, four (Legon, Mudor, Lavender Hill Faecal Sludge Treatment Hill, and Adjen Kotoku) wastewater treatment plants are functional in the Greater Accra Metropolitan Area [19,20]. Municipal sewage management is under the supervision of the Metropolitan and Municipal Assemblies in Ghana. 

The Legon Sewage Treatment plant located in Accra. It can treat 6424 m^3^ of sewage inflow per day and was designed to serve 33,000 residents but currently receives 3606 m^3^ of wastewater per day [12]. In addition to the Achimota hospital, the treatment plant receives sewage from educational institutions (the University of Ghana, Presbyterian Senior High School, Achimota Basic School, University of Professional Studies, and Achimota Senior High School). The treatment plant is made up of a series of waste stabilization ponds made up of three anaerobic, three facultative, and six maturation ponds. The effluents from the plant discharge directly and as a continuous flow into the Onyasia Stream. The Onyasia Stream is one of the tributaries of the Odaw River and runs through five communities in Accra.

### 2.3. Water Sample Collection and Laboratory Analysis and Biochemical Identification

**Figure 2:**
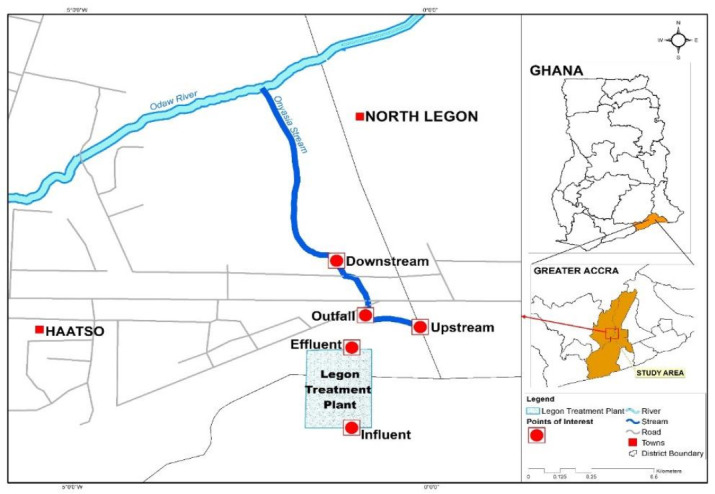
Wastewater and Onyasia stream water sampling sites with reference to the Legon Sewage Treatment plant, Accra, Ghana.

Wastewater and surface water samples were collected (a total of 30 samples) monthly over a six-month period (January–June 2018). Sample collection and analysis were done by the membrane filtration technique according to procedures outlined in the Standard Methods for the Examination of Water and Wastewater, 2012) [21]. Wastewater and stream water samples were collected on a monthly basis from five sampling points (Figure 2): (I) Influent (raw sewage) at the entry point to the sewage treatment plant, (II) Effluent at the exit point of the treatment plant (treated sewage), (III) 500 m upstream from the effluent outfall point into the Onyasia stream, (IV) Sewage outfall point, and (V) 500 m downstream from the outfall point. 

Composite samples for each sampling site were collected and analyzed. Three water samples were systematically collected using an aseptic technique into 500 mL sterile bottles during the morning from each of the five sampling points [21]. All the samples were collected in triplicate and pooled together as one composite sample per sampling location. All samples were immediately transported on ice (within the hour) to the Council for Scientific and Industrial Research-Water Research Institute (CSIR-WRI) microbiology laboratory and analyzed immediately. Water samples were serially diluted by ten-fold serial dilutions in phosphate-buffered saline solution and analyzed using membrane filtration. Bacterial counts were reported as colony-forming units (CFU)/100 mL. Each sample was plated on *Aeromonas* agar base (OXOID, United Kingdom) supplemented with ampicillin for the isolation of *A. hydrophila*, Cetrimide agar (OXOID, United Kingdom) for the isolation of *P. aeruginosa* and Chromo cult coliform agar (MERCK, Germany) for *E. coli*. Inoculated plates were incubated for 24 h at 37 °C for all bacteria. Five presumptive colonies were randomly selected and subjected to Gram strain. This was followed by indole and triple sugar iron agar testing for *E. coli*; oxidase and catalase testing for *A. hydrophila* and *P. aeruginosa*; motility, citrate, and glucose fermentation tests for *A. hydrophila* [22].

Five confirmed isolates were used to perform antibiotic susceptibility testing using the Kirby Bauer Disc Diffusion method, according to Clinical Laboratory Standards Institute (CLSI) guidelines [23]. Zones of inhibition were measured in millimeters and recorded for each antibiotic. 

Antibiotics tested were amoxicillin/clavulanate 20/10 μg, aztreonam 30 μg, imipenem 10 μg, gentamicin 10 μg, tetracycline 30 μg, ciprofloxacin 5 μg, cefuroxime 30 μg, and ceftazidime 30 μg.

### 2.4. Quality Control Procedures

Negative controls were done by plating sterile distilled water. Reference organisms *P. aeruginosa* American Type Culture Collection (ATCC) ATCC 29213 and *E. coli* ATCC 25922 were used as a positive control following 2017 CLSI guidelines.

### 2.5. Study Inclusions and Period

Sewage and Onyasia stream samples were collected from January 2018 to June 2018.

### 2.6. Data Collection, Source of Data, and Validation 

Data variables included identifier and date variables, sewage sample source (influent; effluent) and surface water samples (upstream; outfall; downstream), type of bacteria isolated, bacterial counts, and antibiotic resistance profiles. Information on sample collection points, sample sources, bacterial loads, and resistant profiles were entered from data collection sheets into a laboratory register and then transferred to a database (Microsoft Excel^®^) kept in the laboratory. To ensure data validation, all data in the Microsoft Excel^®^ file were crosschecked with the raw data contained in the laboratory notebook.

### 2.7. Statistical Analysis

Bacterial counts were converted to log_10_ CFU/mL and plotted in graphs for each bacteria every month and by sampling site. These counts and resistance profiles were reported using descriptive statistics. For bacterial counts, the acceptable thresholds set by Ghana Environmental Protection Agency Standards for effluent discharge were for *E. coli* 10 CFU/100 mL and total coliform 400 CFU/100 mL [24]. The Kruskal–Wallis non-parametric was applied to assess mean differences in bacterial counts and the chi-square to assess linear trends. The level of significance was set at *p* ≤ 0.05 and 95% confidence intervals were used where applicable.

## 3. Results

Of the 30 samples taken from each of the five sampling sites (150 in total), *E. coli, A. hydrophila* and *P. aeruginosa* were isolated in all (100%).

### 3.1. Sewage: Bacterial Loads of E. coli, P. aeruginosa, and A. hydrophila

**Figure 3:**
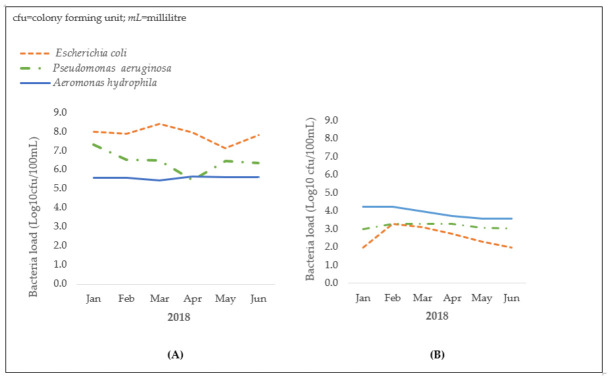
Bacterial loads in influent (**A**) and effluent (**B**) wastewater from the Legon Sewage Treatment Plant, Accra, Ghana (January to June 2018).

Figure 3 shows the monthly bacterial loads (in log_10_ CFU/100 mL) of *E. coli*, *P. aeruginosa*, and *A. hydrophila* in influents and effluents from the Legon sewage treatment plant. There is a considerable decline in bacterial loads in effluents. 

Table 1 compares the bacterial counts of *E. coli*, *P. aeruginosa*, and *A. hydrophila* in influents and effluents from the sewage treatment plant. In effluents, there was a significant 99.9% reduction in mean bacterial load (all three bacteria combined) compared to influents. Significant reductions in mean bacterial load were seen for all the three bacteria tested: *E. coli* (99.9% reduction), *A. hydrophila* (98.8% reduction), and *P. aeruginosa* (99.5% reduction). 

### 3.2. Sewage: Antibiotic Resistance in E. coli, A. hydrophila, and P. aeruginosa in Influent and Effluent Samples

A total of 30 bacterial isolates each for *E. coli*, *A. hydrophila*, and *P. aeruginosa* were tested for antibiotic resistance in both influent and effluent sewage (Table 2 and Table 3). In effluents, antibiotic resistance significantly reduced for tetracycline and ciprofloxacin (*E. coli* and *A. hydrophila*), cefuroxime (*A. hydrophila*), and ceftazidime (*P. aeruginosa*). In contrast, antibiotic resistance increased for gentamicin (*E. coli*), amoxicillin/clavulanate, and imipenem (*A. hydrophila*). 

The highest levels of antibiotic resistance in effluents were for amoxicillin/clavulanate (50% in *E. coli* and 97% in *A. hydrophila*) and aztreonam (33% for *P. aeruginosa*).

### 3.3. Onyasia Stream: Bacterial Counts of E. coli, P. aeruginosa, and A. hydrophila in Water Samples 

**Figure 4:**
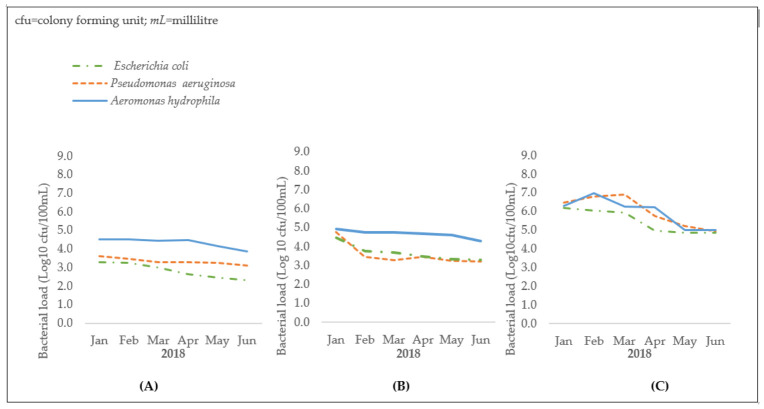
Bacterial loads in upstream (**A**) outfall (**B**) and downstream (**C**) water samples collected from the Onyasia stream, Accra, Ghana (January to June 2018).

Figure 4 gives a graphical representation (in log _10_ cfu/100 mL) of changes in bacterial loads of *E. coli*, *A. hydrophila*, and *P. aeruginosa* in upstream water samples, at the sewage outfall point and downstream water in the Onyasia recipient stream. Going from upstream to downstream, there is a progressive increase in bacterial loads.

Table 4 shows the mean monthly bacterial counts for *E. coli*, *A. hydrophila*, and *P. aeruginosa* in water samples collected upstream, at the sewage outfall point, and downstream in the Onyasia stream. Bacterial counts significantly increased from upstream to downstream. There was a 99.5% overall increase in mean bacterial counts (all three bacteria combined) between upstream and downstream water samples. Between the sewage outfall point and downstream, a 98.8% significant increase was seen predominantly in *E. coli* followed by *P. aeruginosa.*

### 3.4. Onyasia Stream: Antibiotic Resistance in E. coli, A. hydrophila, and P. aeruginosa in Upstream, Outfall and Downstream Water Samples 

Thirty bacterial isolates each for *E. coli*, *A. hydrophila*, and *P. aeruginosa* were tested for antibiotic resistance in upstream, outfall, and downstream water samples (Table 5 and Table 6). There was a progressive increase in antibiotic resistance from upstream, to outfall, to downstream. This trend was significant for five of the seven antibiotics tested in *E. coli*, five of the antibiotics tested for *A. hydrophila*, and three of the five antibiotics tested for resistance in *P. aeruginosa*. In downstream water samples, the highest levels of antibiotic resistance were for amoxicillin/clavulanate (73% in *E. coli* and 80% in *A. hydrophila*), cefuroxime (73% in *E. coli* and 47% in *A. hydrophila*), and in *P. aeruginosa*, aztreonam (53%) and ciprofloxacin (40%).

## 4. Discussion

This is the first study from Ghana that assessed the efficiency of a sewage treatment plant in reducing *E. coli*, *A. hydrophila*, and *P. aeruginosa* counts and their antibiotic resistance patterns, as well as the impact on the recipient stream. The study showed that the Legon sewage treatment plant significantly reduced bacterial counts of *E. coli*, *A. hydrophila*, and *P. aeruginosa* by over 99% in effluents. However, *E. coli* counts in effluent from the treatment plant exceeded the Ghana Environmental Protection Agency’s limits (*E. coli* 10 CFU/100 mL and total coliform 400 CFU/100 mL) for *E. coli* discharged into the environment. In relation to the effluent outfall point in the recipient Onyasia stream, both bacterial counts and antibiotic resistance progressively increased from upstream to downstream and were most marked for *E. coli*, implying intense fecal contamination after the sewage outfall. Depending on the type of bacteria, the highest resistance levels ranged from 33% to 97% for antibiotics listed by WHO to be “highly or critically important for human use” [16]. 

These research findings are of public health importance as they demonstrate the vital role sewage treatment plants can play in protecting the health and livelihoods of communities by reducing bacterial contamination of the environment. Both humans and animals are likely to benefit through reduced incidence of water-borne diseases and reduced acquisition of antibiotic resistance, which is of wider benefit to “One Health” [4]. The study also shows the potential importance of water bodies including urban streams in the emergence and spread of AMR in Ghana. Agricultural produce contaminated by bacteria may get to markets in other cities within Ghana and possibly foreign markets through export. 

The strengths of this study are that all water samples were collected at the same time of day and transported within one hour of collection for laboratory analysis by the same senior laboratory technician to prevent bacterial overgrowth during transit; antibiotic sensitivity testing was performed according to international standards with quality control measures in place [21,23]; the antibiotics tested included those listed by the WHO as being critically (or highly) important in human medicine [16]; and the subject matter addresses an identified national operational research priority for tackling AMR. We also adhered to STROBE (Strengthening the reporting of observational studies in epidemiology) guidelines for the conduct and reporting of this study [25].

The study limitations are that we were unable to assess the effect of seasonal differences due to the six-month study period, and this will become one of the important variables to consider in future research. Antibiotic resistance testing was also limited to three bacteria, but this was a deliberate choice, as the selected bacteria are known to be less fastidious to grow on culture media, and at the same time, they serve as proxies for the contamination of freshwater sources [26]. 

There are some important policy and practice implications. First, the sewage treatment plant was highly efficient in reducing bacterial counts by over 99%—from millions of bacteria to bare hundreds per mL in the effluent. Furthermore, antibiotic resistance levels were lower for tetracycline, ciprofloxacin, cefuroxime, and ceftazidime, which is encouraging, as these antibiotics are commonly used as first- and second-line treatments in humans. However, compared to influents, antibiotic resistance in effluents was higher for gentamicin, amoxicillin/clavulanate, and imipenem. This was not surprising, as it is well established that sewage treatment plants serve as hotspots for the increased transfer of antimicrobial resistance between bacteria [27,28,29]. That said, the 99.9% reduction in bacterial counts in the effluents could imply similar reductions in levels of antibiotic-resistant bacteria, too. Thus, the upside is “less release of resistant bacteria, less contamination, less spread". This finding justifies putting back in action all sewage treatment plants that are currently non-functional in Accra. There have been suggestions to use effluents from sewage plants for irrigation and fish farming in Ghana [30]. Considering that bacterial loads were several million times lower than in the recipient stream water, it would seem logical. A quantitative microbial risk assessment, as well as antibiotic sensitivity testing on agricultural produce from such irrigation schemes, would seem useful to better assess any potential risks associated with such an initiative. 

Second, the progressive and substantial increase in bacterial counts in the Onyasia stream going from the upstream sampling point to the sewage outfall, to the downstream point, all of which were barely 500 meters apart, implies that intense contamination of the stream water is going on. Of concern is that *E. coli*, a fecal pathogen, was most predominant. The Onyasia stream is a very narrow stream with several houses situated along its banks. This suggests other concentrated fecal contamination sources pouring into the Onyasia stream, most likely from household sewage discharge. One would be inclined to think that such contamination would increase exponentially going further downstream as the community density increases. 

The finding of high resistance levels among antibiotics classified by WHO as being “critically important antimicrobials” is of dire concern. For example ciprofloxacin are used for the treatment of urinary tract infections and essential second-line drug agents for multidrug-resistant tuberculosis [31]. Carbapenems (e.g., imipenem) and monobactams (e.g., aztreonam) are last-resort antibiotics for the treatment of multidrug-resistant Enterobacteriaceae and *P. aeruginosa* infections in humans, most of which occurs in intensive care units. Severely ill COVID-19 patients with pneumonia depend on such life-saving antibiotics [31]. 

The study findings call for immediate and medium-term measures that could be summarized as “Inform, Educate, Protect, and Act”. The immediate measures would include informing and engaging with communities to enhance their awareness of the non-potable nature of the Onyasia stream water; educating communities that water must be boiled before household use; educating consumers of vegetables and particularly salads that are grown alongside the Onyasia stream that these products should be washed with pipe-borne water and then disinfected with vinegar or mild chlorine to destroy *Enterobacteriaceae* spp. [32]; and protecting farmers through the use of protective wear (e.g., gloves and gumboots) to reduce direct contact with contaminated water. This is important as farmers are prone to cuts and wounds that could be rapidly colonized by highly resistant *P. aeruginosa*, resulting in chronic and debilitating disease particularly in those with reduced immunity [33]. 

There are also medium-term measures upon which one could act. For example, farmers could be assisted in adopting irrigation methods that reduce or eliminate contact of irrigation water with the green vegetables i.e., through the use of drip or furrow methods [34]. 

Reducing fecal contamination of the Onyasia stream is essential, as it would improve and preserve the quality of downstream water used by communities. Since the Legon sewage treatment plant is currently functioning at roughly 30% capacity, there is ample space to explore the feasibility of linking up households in the area to the Legon sewage influent pipe system. 

Ensuring that each household has a septic tank might also be an interim and alternative measure to be considered. Other considerations would include increasing the numbers of sewage treatment plants in Accra and establishing legislation to prevent anarchic discharge of household sewage into the Onyasia stream and other such water bodies. We suggest deliberations at the One Health AMR coordinating committee in Ghana, so that material and political resources can be galvanized to consider these and other mutually complementary actions. There is an urgent need to move any rhetoric to action.

## 5. Conclusions

In conclusion, this study has highlighted the important role that a sewage treatment plant can play in reducing bacterial contamination of the environment. However, antibiotic-resistant bacteria may remain in treated wastewater. In addition, high bacterial counts and antibiotic-resistant bacteria in the Onyasia stream show the potential role water bodies such as the Onyasia stream can play in the spread of water-borne diseases and the wider transmission of antimicrobial resistance. 

## Figures and Tables

**Table 1:** Bacterial counts in influent and effluent samples from the Legon Sewage Treatment plant, Accra, Ghana (January to June 2018).

Sample	*E. coli*		*A. hydrophila*		*P. aeruginosa*	
	(Mean cfu/100 mL)	*p*-Value ^1^	(Mean cfu/100 mL)	*p*-Value ^1^	(Mean fu/100 mL)	*p*-Value ^1^
**Influent**	102,266,667	<0.01	376,333	<0.001	5,666,667	0.01
**Effluent**	710		9603		1550	

^1^ Kruskal–Wallis test (comparing influent and effluent).

**Table 2:** Antibiotic resistance of *E. coli* and *A. hydrophila* isolates in influent and effluent wastewater from the Legon Sewage Treatment Plant, Accra, Ghana (January to June 2018).

Antibiotics	Isolates Resistant to Antibiotics					
	*E. coli*			*A. hydrophila*		
	Influent (N = 30)	Effluent (N = 30)	*p*-Value ^1^	Influent (N = 30)	Effluent (N = 30)	*p*-Value ^1^
	n (%)	n (%)		n (%)	n (%)	
Gentamicin 10 μg	2(7)	8 (27)	0.04	4(13)	5(17)	0.5
Amoxicillin/Clavulanate 20 μg	15(50)	15(50)	0.5	18(60)	29(97)	<0.001
Tetracycline 30 μg	24(80)	11(37)	<0.001	27(90)	7(23)	<0.001
Ciprofloxacin 5 μg	19(63)	3(10)	<0.001	20(67)	4(13)	<0.001
Imipenem 10 μg	1(3)	5(17)	0.1	3(10)	10(33)	0.03
Cefuroxime- 30 μg	15(50)	14(47)	0.5	22 (73)	15(50)	<0.001
Aztreonam 30 μg	9(30)	6(20)	0.3	12(40)	11(37)	0.5

^1^ Chi-square test.

**Table 3:** Antibiotic resistance of *P. aeruginosa* in influent and effluent wastewater from the Legon Sewage Treatment Plant, Accra, Ghana (January to June 2018).

Antibiotics	Isolate Resistance to Antibiotics		
	*P. aeruginosa*		
	Influent (N = 30)	Effluent (N = 30)	*p*-Value ^1^
	n (%)	n (%)	
Gentamicin 10 μg	2(7)	5(17)	0.4
Ciprofloxacin 5 μg	9(30)	4(13)	0.2
Imipenem 10 μg	1(3)	2(7)	0.5
Aztreonam 30 μg	9(30)	10(33)	0.5
Ceftazidime 30 μg	6(20)	1(3)	0.02

^1^ Chi-square test.

**Table 4:** Bacterial loads in upstream, sewage outfall, and downstream water samples collected from the Onyasia stream, Accra, Ghana (January to June 2018).

Sample ID	*E. coli*		*A. hydrophila*		*P. aeruginosa*	
	(Mean cfu/100 mL)	*p* Value ^1^	(Mean cfu/100 mL)	*p* Value ^1^	(Mean cfu/100 mL)	*p* Value ^1^
Upstream	955	0.01	2350	0.03	24,433	0.05
Outfall	11,900		8033		52,233	
Downstream	3,043,333		64,100		2,536,667	

^1^ Kruskal–Wallis test (comparing upstream and downstream).

**Table 5:** Antibiotic resistance profiles of *E. coli* and *A. hydrophila* isolates in upstream, sewage outfall, and downstream of the Onyasia stream, Accra, Ghana (January to June 2018).

Antibiotics	Isolates Resistant to Antibiotics							
	*E. coli*				*A. hydrophila*			
	Upstream (N = 30)	Outfall (N = 30)	Downstream (N = 30)	*p*-Value ^1^	Upstream (N = 30)	Outfall (N = 30)	Downstream (N = 30)	*p*-Value ^1^
	n (%)	n (%)	n (%)		n (%)	n (%)	n (%)	
Gentamicin 10 μg	1(3)	6(20)	8(27)	0.03	0(0)	2(7)	3(10)	0.2
Amoxicillin/Clavulanate 20 μg	14(47)	18(60)	22(73)	0.03	8(27)	23(77)	24(80)	0.2
Tetracycline 30 μg	10(30)	9(50)	17(40)	0.41	3(10)	4(13)	10(33)	<0.001
Ciprofloxacin 5 μg	3(10)	9(30)	10(33)	0.05	0(0)	4(13)	5(17)	0.05
Imipenem 10 μg	0(0)	4(13)	7(23)	0.005	0(0)	5(16)	8(27)	<0.001
Cefuroxime 30 μg	12(40)	15(50)	22(73)	0.01	2(7)	11(37)	14(47)	<0.001
Aztreonam 30 μg	4(13)	8(27)	10(33)	0.06	0(0)	5(17)	12(40)	<0.001

^1^ Chi-square for linear trend using upstream as a baseline.

**Table 6:** Antibiotic resistance profiles of *P. aeruginosa* isolates in upstream, outfall, and downstream of the Onyasia stream, Accra, Ghana (January to June 2018).

Antibiotics	Isolates Resistant to Antibiotics			
	*P. aeruginosa*			
	Upstream (N = 30)	Outfall (N = 30)	Downstream (N = 30)	*p*-Value
Gentamicin 10 μg	1(3)	6(20)	8(27)	0.02
Ciprofloxacin 5 μg	1(3)	7(22)	12(40)	<0.01
Imipenem 10 μg	0(0)	1(3)	2(7)	0.2
Aztreonam 30 μg	6(20)	15(50)	16(53)	0.01
Ceftazidime 30 μg	1(3)	4(13)	4(13)	0.2

^1^ Chi-square for linear trend using upstream as a baseline.

## Data Availability

The data presented in this study are available on request from the corresponding author.
